# A Reconfigurable, Nonlinear, Low-Power, VCO-Based ADC for Neural Recording Applications

**DOI:** 10.3390/s24196161

**Published:** 2024-09-24

**Authors:** Reza Shokri, Yarallah Koolivand, Omid Shoaei, Daniele D. Caviglia, Orazio Aiello

**Affiliations:** 1 Biomedical Integrated Systems Lab, University of Tehran, Tehran 1439957131, Iran; oshoaei@ut.ac.ir; 2 Dipartimento di Ingegneria Navale, Elettrica, Elettronica e delle Telecomunicazioni (DITEN), University of Genoa, 16145 Genoa, Italy; daniele.caviglia@unige.it (D.D.C.); orazio.aiello@unige.it (O.A.); 3 Department of Electronics, Khajeh Nasir Toosi University of Technology, Tehran 1631714191, Iran; y.koolivand@kntu.ac.ir

**Keywords:** neural recording systems, VCO-based ADC, nonlinear quantization, parabolic function ADC

## Abstract

Neural recording systems play a crucial role in comprehending the intricacies of the brain and advancing treatments for neurological disorders. Within these systems, the analog-to-digital converter (ADC) serves as a fundamental component, converting the electrical signals from the brain into digital data that can be further processed and analyzed by computing units. This research introduces a novel nonlinear ADC designed specifically for spike sorting in biomedical applications. Employing MOSFET varactors and voltage-controlled oscillators (VCOs), this ADC exploits the nonlinear capacitance properties of MOSFET varactors, achieving a parabolic quantization function that digitizes the noise with low resolution and the spikes with high resolution, effectively suppressing the background noise present in biomedical signals. This research aims to develop a reconfigurable, nonlinear voltage-controlled oscillator (VCO)-based ADC, specifically designed for implantable neural recording systems used in neuroprosthetics and brain–machine interfaces. The proposed design enhances the signal-to-noise ratio and reduces power consumption, making it more efficient for real-time neural data processing. By improving the performance and energy efficiency of these devices, the research contributes to the development of more reliable medical technologies for monitoring and treating neurological disorders. The quantization step of the ADC spans from 44.8 mV in the low-amplitude range to 1.4 mV in the high-amplitude range. The circuit was designed and simulated utilizing a 180 nm CMOS process; however, no physical prototype has been fabricated at this stage. Post-layout simulations confirm the expected performance. Occupying a silicon area is 0.09 mm^2^. Operating at a sampling frequency of 16 kS/s and a supply voltage of 1 volt, this ADC consumes 62.4 µW.

## 1. Introduction

Treatments to deal with neurological disorders are usually limited and to seek relief, and many patients explore complementary and alternative medicine [1]. Innovative non-pharmacological approaches, such as neuro-stimulation, are gaining significance for addressing some of the most widespread and challenging neurological disorders. In particular, brain stimulation stands out as the predominant surgical approach for treating movement disorders. It exhibits potential in conditions like epilepsy, neuropsychiatric disorders, memory issues, chronic pain, and traumatic brain injury, with continually emerging applications [2,3,4,5,6].

Open-loop neuro-stimulators, lacking real-time feedback from the stimulated brain, can result in potentially unintended adverse outcomes [3,7]. On the other hand, a closed-loop neuro-stimulator provides real-time feedback and can adapt or adjust its treatment according to the individual’s response. In this way, some undesirable effects, such as the risk of overstimulation, limited precision, inefficient power consumption, adaptation challenges, and safety concerns can be prevented [4,8,9].

Closed-loop neuro-stimulators consist of three main components: a recording unit; a stimulation unit; and a control system, which includes the data processing unit and wireless data and power telemetry systems. The recording unit typically comprises a low-noise amplifier and an analog-to-digital converter (ADC) [10]. In some cases, a multiplexer is included to facilitate channel selection, particularly in high-density neural recording systems [11]. It is noteworthy that relying on high dynamic range ADC to eliminate low-noise amplifiers has been explored in recent research [12].

In most of applications like neuro-prosthesis and brain–computer interfaces, the data after the recording must be sent to the main processor outside the body. For compatibility with the wireless telemetry system’s limited bandwidth, some techniques such as spike detection, spike sorting, and digital processing and compression have been adopted. Spike sorting and spike detection are techniques suitable for a wide variety of neuro-prosthetic applications. Signal processing and compression techniques have successfully been proven in terms of data compression; however, they face challenges regarding circuit implementation efficiency, particularly in terms of silicon area and power dissipation, especially if a large number of recording channels is required [13]. Recent developments in the design of signal-specific ADCs involve innovative concepts focused on nonlinear quantization to emphasize regions where more information is concentrated in the amplitude domain. These types of ADCs aim to address the bottleneck of digital back-end power consumption by reducing the volume of data flowing into the digital domain. This is achieved by (a) extracting sensory features in the analog domain and digitizing these features instead of uniformly quantizing the raw data and (b) employing data compression techniques through analog non-linearities to decrease the length of the word required for the specific task [13,14,15,16,17,18,19,20,21].

**Figure 1:**
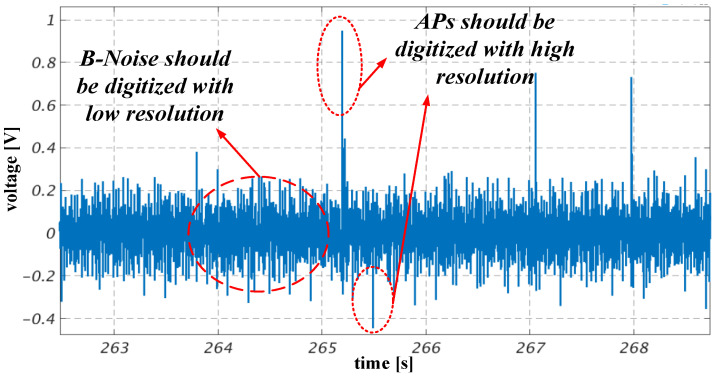
Neural signal illustrating action potentials (APs) and background noise obtained from a rat sample.

Figure 1 illustrates a neural signal recorded from a live rat’s brain. This signal was recorded by the Neurovision electrophysiological signal recorder of the Niktek Company in the Biomedical Integrated Systems Lab, University of Tehran [22]. During periods of neuronal inactivity, the recorded signal captures only the background noise (B-noise). In contrast, the excitation phase reveals prominent spikes known as action potentials (APs). In neuroscientific studies and neuro-prosthetic applications, APs convey essential information from cell to cell, and their sequences allow us to extract valuable insight into brain activities. While single-neuron firings encode neural information in the time domain, the amplitude of APs enhances spatial resolution and aids in distinguishing between APs originating from different neurons in high-density single-unit recordings.

Typically, implantable neural recording systems employ linear ADCs, resulting in the digitization of non-useful B-noise with the same resolution as valuable APs. Given that neurons are often at rest, a substantial portion of the outgoing bit-rate is squandered on transmitting noise content within the neural signal. Nonlinear ADCs present a promising technology for enhancing the performance of implantable neural recording systems. By minimizing the bit-rate wasted on noise, nonlinear ADCs can enhance the signal-to-noise ratio (SNR) of the digitized neural signal, concurrently reducing power consumption and the area occupied by the ADC. These nonlinear ADCs offer advantages in applications with limited bandwidth in wireless telemetry, such as implantable brain–machine interfaces featuring high-density microelectrodes. Additionally, despite advancements in reducing the power consumption of analog-to-digital converters (ADCs), the power consumed by digital backend processing remains dominant in numerous portable always-on and multi-sensor systems. Nonlinear ADCs further provide the benefit of decreasing the volume of data streaming into the digital backend [13,14,15,16,17,18,19,20,21].

In this paper, a novel reconfigurable, nonlinear, voltage-controlled oscillator (VCO)-based ADC is presented. It exploits the nonlinear properties of metal oxide semiconductor field effect transistor (MOSFET) varactors. The structure of the paper is outlined as follows: Section 2 provides a brief overview of nonlinear neural recording ADCs and the traditional VCO-based ADCs. The newly proposed nonlinear ADC is introduced in Section 3. Section 4 shows the post-layout simulation results, and conclusions are drawn in Section 5.

## 2. Brief Review of the State of the Art

### 2.1. Nonlinear Signal-Specific ADCs

Most of the nonlinear signal-specific ADCs [13,20] use successive approximation register (SAR) architecture. One way to implement a nonlinear (e.g., logarithmic or exponential) SAR ADC is to use the standard linear SAR ADC structure but replace the linear charge redistribution digital-to-analog converter (DAC) with a nonlinear counterpart. These nonlinear SAR ADCs operate similarly to their linear counterparts. The comparator compares the analog input voltage with threshold voltage levels from the specific weighted DAC to sequentially determine the digital output from the most significant bit (MSB) to the least significant bit (LSB). For example, the article [13] introduces an 8-bit two-step SAR ADC utilizing a piece-wise linearly approximated exponential quantization function for recording neural signals. Similarly, the article [20] presents a nonlinear quantization technique specifically designed for biomedical signal processing. The architecture that is presented in [16] features a two-stage conversion process with a programmable coarse-conversion stage and a fixed fine-conversion stage. The first stage uses a programmable, thermometer-encoded capacitive digital-to-analog converter (CDAC) to define coarse segments, which can be adjusted to support non-linear transfer functions. The second stage performs fine conversion within these segments, resulting in variable resolution across the full-scale range. This method optimizes the resolution in selected regions while reducing the total number of capacitors needed, leading to efficient use of area and resources.

The logarithmic level-crossing ADC presented in [17] shows a fixed comparison window that includes two comparators, a charge-sharing logarithmic DAC, control logic, an up–down counter, and DC voltage sources. Reference [18] employs a diode-connected CMOS transistor powered by a binary-weighted current DAC. However, the current fluctuates within a designated range to ensure that the output voltage (the drain-source voltage of the diode-connected device) is intentionally kept below the transistor threshold voltage (Vth) and ideally exceeds 4V_T_, where V_T_ represents the thermal voltage, and this logarithmic DAC can be used as the core of exponential ADC.

The design presented in [14] aims at optimizing data encoding by maximizing weighted entropy or minimizing code size while maintaining information conservation. The key innovations include the development of highly non-linear, adaptable mapping functions for different signal distributions; the implementation of an iterative, resource-efficient on-chip mapping process; and improvements upon existing non-linear encoding techniques by introducing fully configurable non-linear data compression, integrated into a SAR-like ADC.

### 2.2. VCO-Based ADC

The supply voltage scaling aimed at reducing power consumption significantly impacts the performance of traditional voltage-based ADCs, as their dynamic range and speed are inversely proportional to the supply voltage. As a result, designing conventional voltage-based ADCs becomes progressively more and more challenging, leading to a preference for time-based ADCs in various applications within these processes [23,24,25,26,27,28,29,30,31].

**Figure 2:**
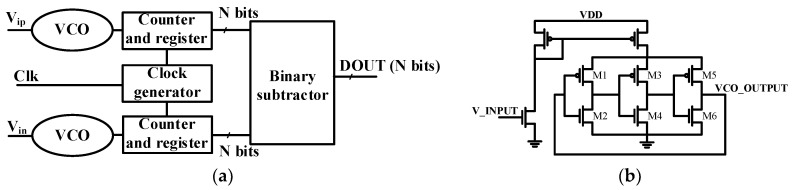
(**a**) General VCO-based ADC architecture; (**b**) schematic of the general VCO units.

The voltage-controlled oscillator (VCO) serves as the vital component of the VCO-ADC. Essentially, the VCO transforms the value of the input voltage into a periodic signal whose frequency depends on the average of the input. The VCO’s output acts as a clock signal for a counter, as depicted in Figure 2. This counter tallies the number of clock edges within a fixed sampling gate. At the end of each sampling interval, the counter outputs a digital code representing the input signal, forming what is known as a VCO-based ADC. The residual phase of the VCO output at the end of each sampling cycle carries over as the initial phase for the subsequent period, resulting in first-order noise shaping for VCO-based ADCs. To successfully deploy a VCO-based ADC, the difference between the maximum and minimum frequencies must surpass 2^N^ × F_s_, where F_s_ is the sampling frequency and N represents the nominal number of the ADC bits. This requirement ensures that the frequency range covered by the VCO accommodates the necessary granularity for accurately representing the input signal across the specified bit resolution. In practical terms, the dynamic range of the VCO, represented by the frequency difference, needs to be sufficiently wide to capture the entire spectrum of input signals with the desired precision.

Two parameters influence the frequency of the ring VCO: the current of the VCO and the load capacitor of the delay cells [23]. The ADC structure described in [32,33,34,35] comprises a current mirror and a ring oscillator. The analog input voltage is firstly converted into a current that feeds the current-starved inverters in the ring VCO, influencing the oscillator’s output frequency. Such works employ a differential architecture to enhance the ADC linearity and a subtractor to subtract the register contents of the positive and negative inputs.

## 3. Proposed Architecture

**Figure 3:**
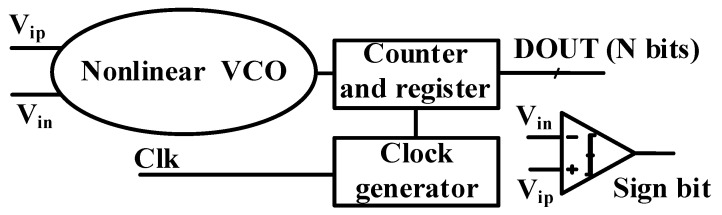
Block diagram of the proposed architecture.

The proposed nonlinear VCO-based ADC is depicted in Figure 3. In contrast to conventional designs, the proposed architecture eliminates one VCO, as well as the counter/register and the subtractor components.

Additionally, the differential input signal is solely applied to the PMOS varactors (the capacitor loads of the delay cells within the VCO). The nonlinear variability of the frequency generated by the VCO as a function of the input voltage value is obtained by adding two p-channel varactors to each internal node of the inverter loop.

**Figure 4:**
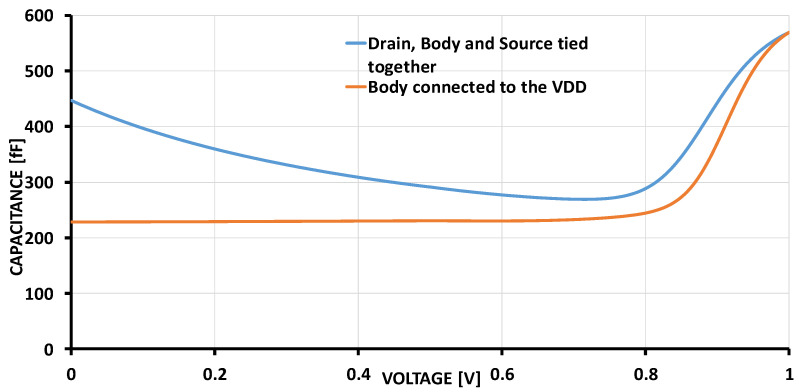
Capacitance of two PMOS transistors, the bulk of which are connected to source and drain (blue line) or VDD (orange line).

Figure 4 illustrates the capacitance characteristics of the two PMOS transistors regarding the input signal. The transistor gates are biased at half the supply voltage (VDD/2), while the drain and source are shorted together and connected to the input. In case (a—blue line), the bulk is tied to both the source and drain, while in case (b—orange line), the bulk is connected to the supply voltage VDD. In case (a), the transistor bulk undergoes both accumulation and inversion regimes at low and high input voltages, respectively, while in case (b), the bulk remains in constant inversion mode—weak inversion at low input and strong inversion once the threshold is reached [36,37,38]. Consequently, the capacitance behaviors are similar in both cases at high input level, but they differ for low-level input signals. Since case (b) exhibits nearly constant capacitance at low input signals, it is selected for the proposed nonlinear ADC to effectively suppress B-noise in this application. The width and length of both PMOS are 11 um and 11 um, respectively.

**Figure 5:**
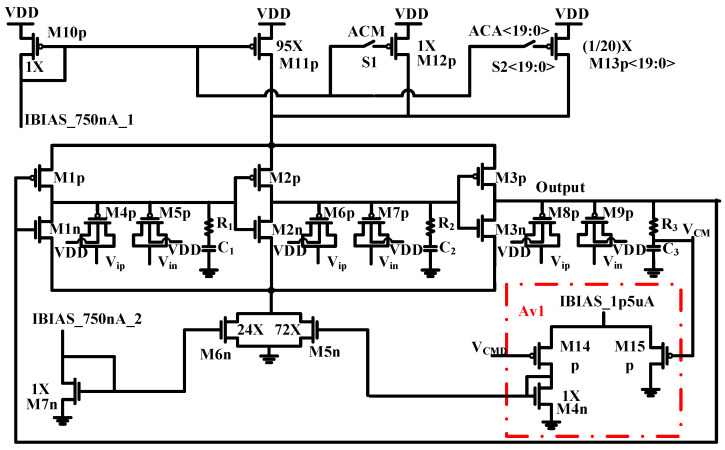
Schematic of the proposed VCO-based ADC.

Figure 5 presents the simplified VCO circuit employed as the core of the nonlinear ADC. In the proposed VCO-based ADC, the delay cells consist of a simple inverter (M1p-M1n, M2p-M2n, M3p-M3n), with its capacitive load consisting in a PMOS varactor pair (M4p-M5p, M6p-M7p, M8p-M9p). As depicted in Figure 5, in each cell, both varactors are connected with their gates to the output node, while their drain/source terminals are connected to the positive input voltage, Vip, and to the negative input, Vin, respectively, to maintain symmetry.

The ring active nodes are biased to VDD/2 through the tail current source M5n-M6n and by using the common mode feedback circuit (Av1); the bulk of the varactors is connected to the highest available voltage, VDD.

**Figure 6:**
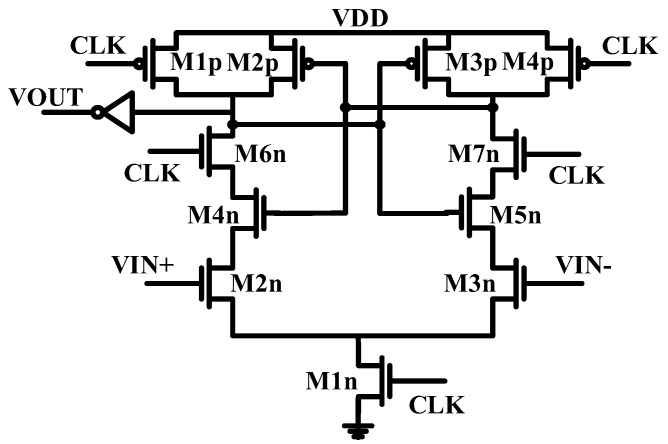
Schematic of the dynamic sign detection comparator.

A dynamic comparator is used to detect the sign of the signal after the conversion process (Figure 6). Based on the application (neuro-prostheses), the data will be processed outside of the chip. Therefore, to simplify the digital design and minimize area and power consumption, we chose to detect the sign of the digital code by adding a leading sign bit. This approach allows for a more efficient implementation compared to other coding schemes, such as two’s complement, which would increase complexity without providing significant advantages for this specific use case. The proposed topology effectively eliminates B-noise. In contrast, the offset of the comparator has minimal impact on the ADC’s performance. Notably, the frequency variation of the VCO around the zero input is negligible, effectively removing the B-noise. Expanding the quantization level around the zero point can ease the design of the sign detection comparator against mismatches.

The dynamic comparator is represented in Figure 6. Due to the wide step around the zero point, the mismatch and offset of the comparator are negligible, and the sign detection comparator poses no serious challenges in circuit design. It should be noted that branches composed of a series of a high resistor and a capacitor (R1–R3, C1–C3) are employed at the output nodes of the delay cells of the VCO as low-pass filters to sense the DC voltages of the nodes. These DC voltages serve as input to the difference amplifier, Av1, setting the bias voltage of the output nodes of the delay cells of the VCO to VDD/2. By utilizing this functionality, the DC voltage of Av1 can be modified, thereby altering the nonlinear curve of the VCO-based ADC under consideration. This capability introduces reconfigurability to the ADC, enhancing its versatility and adaptability. MOS capacitors are sensitive to mismatches, process corners, and temperature variations. To mitigate mismatch issues, transistor sizes are chosen to be as large as possible. However, in VCO-based ADCs, the difference between the maximum and minimum frequency of the VCO must be greater than 2^N^ times the sampling frequency to achieve the desired resolution, which is a bounding limit for enlarging the MOS capacitors. The mismatch variations could be compensated using an appropriate calibration scheme, as mentioned in Figure 5. Based on the Monte Carlo simulations, the current source/sink is identified as the most vulnerable part, particularly sensitive to mismatch. To keep these variations under control, the transistor M12p and the array of transistors M13p<19:0> are added and are used for calibration. Thus, the frequency variation can be reduced to less than half of the sampling frequency. These transistors can be turned on or off by switch S1 or the array of switches S2<19:0>. These switches are transmission gates constructed of PMOS and NMOS transistors in parallel. Based on Monte Carlo simulations in the simulation section, it was found that the main contributor to mismatch error is the current source/sink. The current sink has a variable component that adapts to the constant current source. Therefore, the calibration mechanism is applied to the current source. Variations in the current source or sink only affect the offset frequency of the VCO, and the frequency waveform is determined by MOS capacitors, whose mismatch can be considered negligible. Half of VDD is applied to all ADC inputs, and after sending the data to the output by toggling the switches (increasing and decreasing currents), the offset frequency is set to the same value for all ADCs. The sigma value of the ADCs, based on Monte Carlo simulations, is about 30 kHz, with an offset frequency of around 19.8 MHz. The current source value is 96X, which can be adjusted to 1/20 of X (by S2 switch arrays) for calibration purposes.

**Figure 7:**
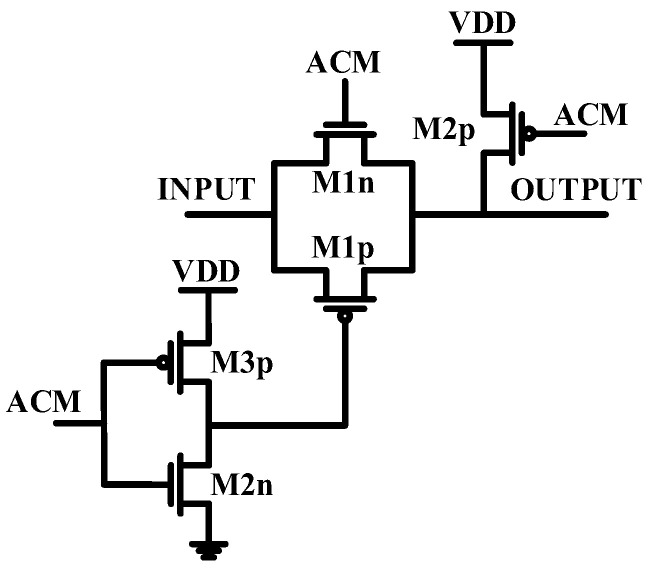
Schematic of the switches for the calibration part.

Despite the mismatch errors, corner variation is not random and is consistent across all the ADC channels on a chip. Therefore, an additional ADC is implemented to calibrate the main ADCs. The calibration ADC is activated during chip startup, being exposed to a slow ramp signal. The resulting output codes are transmitted to an external host, creating a lookup table for calibrating the main ADCs. Although area and power are severe constraints for high-density neural recording systems, they are not critical for the external host processor. Matching the received code with that of the lookup table and performing mathematical operations on the host side can be done without major concerns about power and area. A single ramp generator is used for calibration to eliminate mismatch effects. This generator is applied to the ADC inputs during the calibration phase. It consists of a simple charge pump, which charges a capacitor with a low current, and an amplifier to drive all the ADCs. Since the ramp generator is shared across the entire chip, it is not considered in the layout. The step width at higher amplitudes is 1.4 mV, and 1/8 of this step width is used to define how slowly the ramp generator operates, with a slope of 0.175 mV per 40 µs. As the ADC is symmetric around half of the VDD, only half of the full scale is swept. The total calibration time is 2858 × 40 µs. It is important to note that the main ADC is susceptible to the common mode signal, and the preceding stage (amplifier/buffer) should provide a differential signal over VDD/2 as the common mode signal. The transistor dimensions are provided in Table 1. As for the RC series low-pass filters, the values used are 500 kΩ and 100 fF, respectively. The transmission gate schematic is illustrated in Figure 7. Both the switch S1 and the switch array share the same basic structure, but their transistor sizing differs. The specific dimensions of the switches are detailed in Table 2.

**Figure 8:**
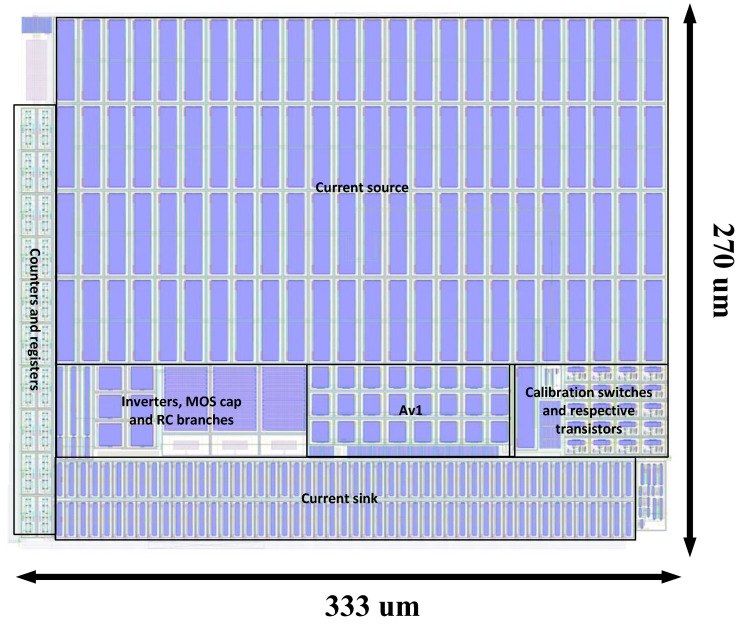
Layout of the proposed ADC.

The layout of the proposed VCO-based ADC was drawn in the TSMC 180 nm CMOS technology node is and reported in Figure 8. The primary components sensitive to process asymmetries are the current source and current sink. To mitigate these effects, we implemented a common centroid layout for these components, ensuring proper matching between the reference current and the output currents, which directly influence the VCO’s performance. As an example, in the current source, all transistors (e.g., X95) are symmetrically placed around the reference transistor of the current mirror to optimize matching. The corresponding components are labeled in the figure for clarity.

## 4. Simulation Results

**Figure 9:**
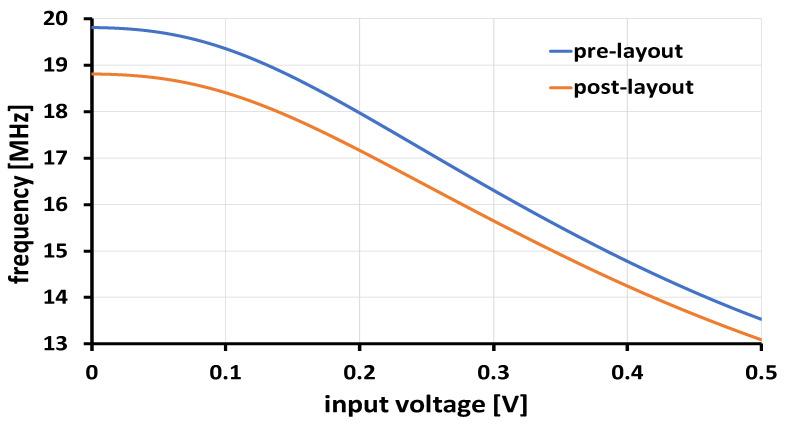
Nonlinear VCO-based ADC frequency variation vs. input voltage (the orange (blue) waveform represents the post (pre)-layout simulation results).

All parasitics were considered in the post-layout simulation. The layout was extracted using Calibre, and both resistive (R) and capacitive (C) parasitics were included in the simulations for the proposed architecture. Post-layout simulations were performed on the proposed nonlinear VCO-based ADC operating at 1 V supply voltage. The VCO’s output frequency ranges from 13.5 MHz to 19.82 MHz, corresponding to a differential input signal variation from −1 V to 1 V. Figure 9 illustrates the frequency variation for the differential voltage component V_diff_, where V_ip_ = 0.5 + V_diff_ and V_in_ = 0.5 − V_diff_. Since the ADC waveform is symmetric around the y-axis, the frequency variation is shown for V_diff_ ranging from 0 V to 0.5 V. The orange (blue) waveform represents the pre(post)-layout simulation results. Both the curves meet the performance criteria of an 8-bit 16 kS/s ADC.

**Figure 10:**
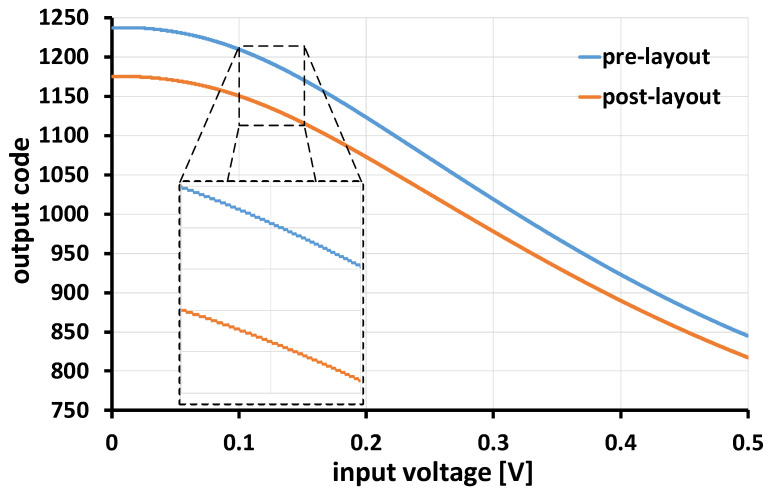
Nonlinear VCO-based ADC transfer curve (the orange (blue) waveform represents the post (pre)-layout simulation results).

**Figure 11:**
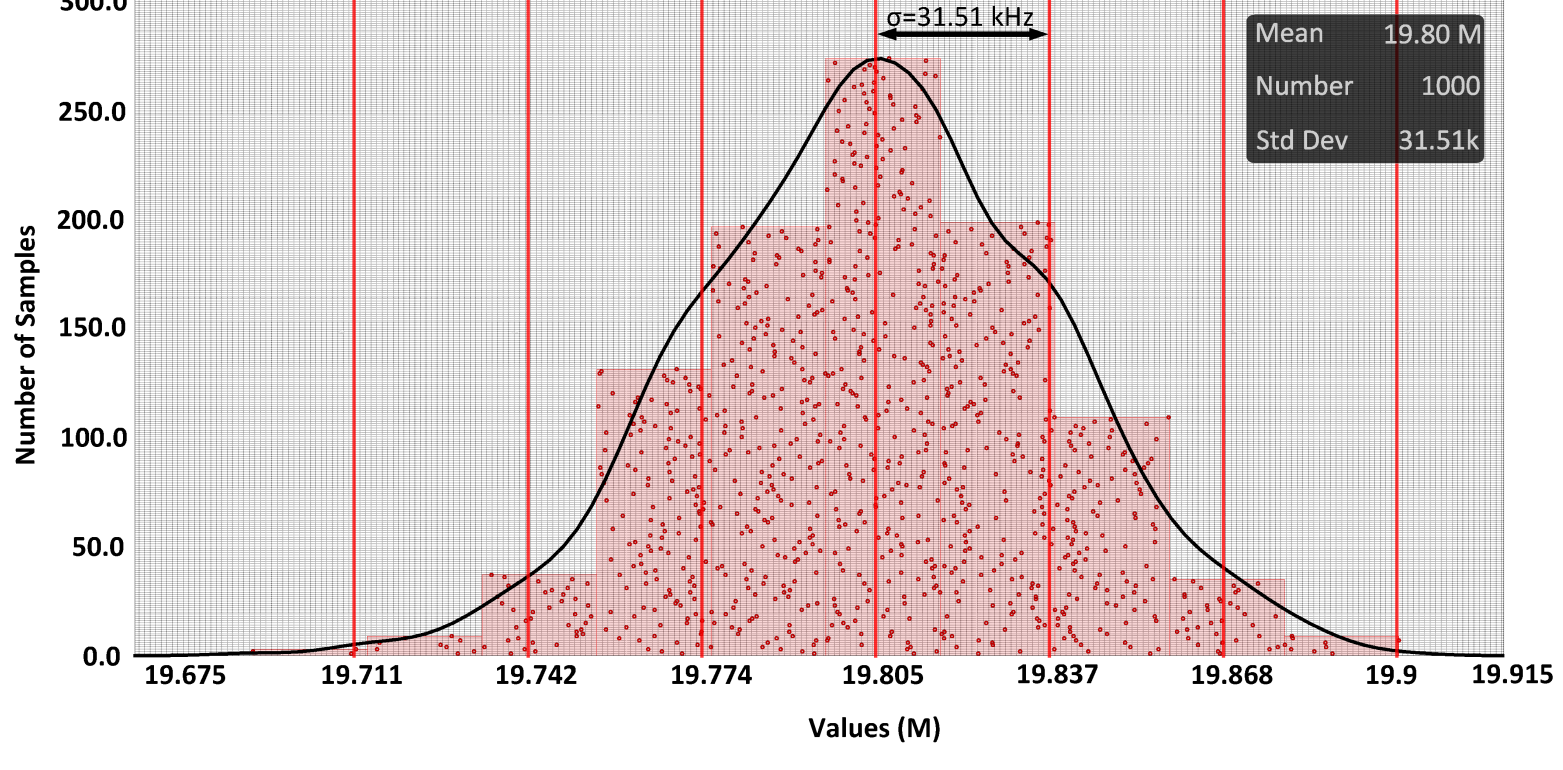
Monte Carlo simulation results considering mismatch models for all the components (mean = 19.80 MHz, standard deviation = 31.51 kHz).

The respective ADC transfer curve is shown in Figure 10, where the black line represents the input ramp voltage. The widths of the first and last codes in the schematic (post-layout) simulation were 51.6 mV (44.8 mV) and 1.2 mV (1.4 mV), respectively, after removing the LSB bit of the counter. Background noise was reduced by increasing the step width of the initial steps. The step width at higher amplitudes was 1.4 mV, while in the first step (zero), it was 44.8 mV. The noise reduction was approximately 20 log (44.8/1.4), which was equal to 30.1 dB. The difference between the post-layout and schematic results can be corrected using a look-up table created by offline calibration. The ADC can effectively eliminate background noise. The ADC’s power consumption, obtained from simulations at the highest oscillation frequency (V_in_ = V_ip_ = VDD/2), was 62.4 µW. Results of the Monte Carlo simulations are depicted in Figure 11, incorporating a mismatch model for all components across 1000 runs. The observed mismatch error (standard deviation) was 31.5 kHz for V_in_ = V_ip_ = VDD/2, surpassing the sampling frequency. The mismatch error ratio to base frequency was 0.16%.

**Figure 12:**
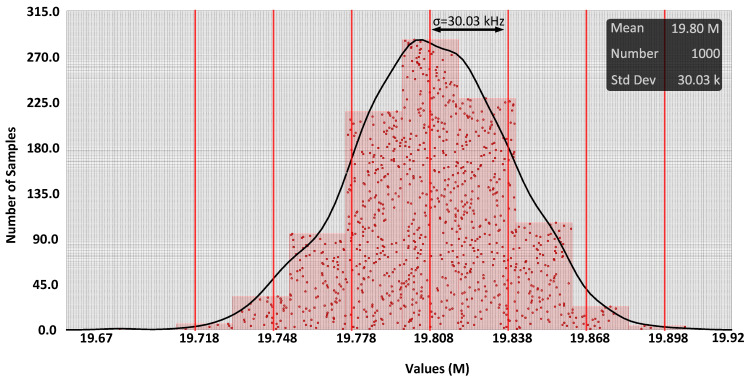
Monte Carlo simulation results considering mismatch models for current mirrors and the common mode feedback amplifier (mean = 19.80 MHz, standard deviation = 30.03 kHz).

Additionally, Figure 12 displays a repetition of the Monte Carlo simulation, in this case accounting for just the current mirrors and the common mode feedback amplifier. The standard deviation was 30.03 kHz for V_in_ = V_ip_ = VDD/2. Notably, all errors were associated with the current mirrors. On this basis, a calibration circuit to adjust the current is needed. For implementing the calibration, parallel current mirrors with a significantly low current ratio to the main current mirrors should be considered, as shown in Figure 5. Their activation depends on the base frequency, wherein they should be switched on or off provided that the base frequency error remains below 12.5 kHz (half of the least significant bit, LSB).

**Figure 13:**
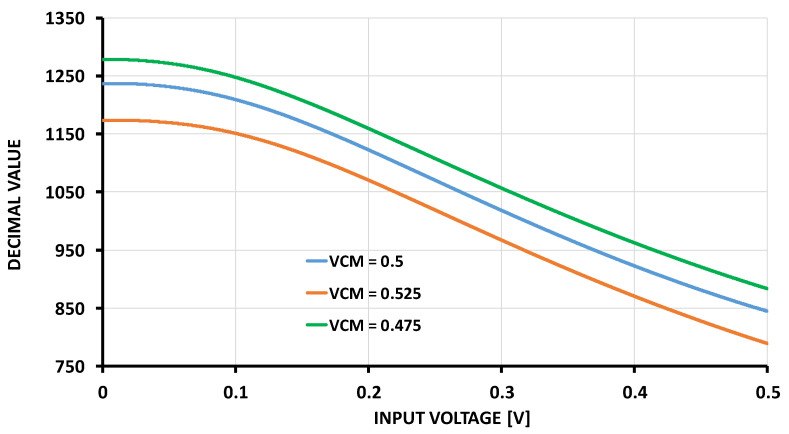
Simulation results for different DC voltages of the Av1.

To support the claim regarding the reconfigurability of the VCO-based ADC and its associated benefits, we conducted simulations to analyze the effect of the DC voltage applied to AV1 on the non-linearity of the ADC. The simulation results in Figure 13 show that varying the DC voltage of AV1 significantly influenced the first step of the ADC. Specifically, for AV1 values of 0.475, 0.5, and 0.525, the variations in the first step were 38.8 mV, 51.6 mV, and 54.8 mV, respectively. In contrast, all higher step values exhibited no variation. These results demonstrate that the DC voltage adjustment allows for reconfigurability, particularly at lower steps, improving the ADC’s adaptability to different signal conditions.

Process variations were considered during the initial offline calibration. Additionally, since the circuit is designed for implantable devices, we assumed a constant ambient temperature of 37 °C. As for voltage variations, the circuit exhibited sensitivity to such changes, which can be compensated for using an additional calibration circuit.

Table 3 provides a summary of the performance of the proposed recording system in comparison to some prior art implementations.

## 5. Conclusions

This paper introduces a novel nonlinear analog-to-digital converter (ADC) specifically designed for recording neural signals in biomedical applications, with a focus on spike sorting. The ADC effectively reduces background noise by leveraging the nonlinear functionality of MOSFET varactors and voltage-controlled oscillators (VCOs). Unlike conventional VCO-based ADCs, this system modulates the capacitance of PMOS varactors to nonlinearly adjust the VCO frequency, achieving a parabolic quantization function. This function digitizes low-amplitude noise with coarse resolution while capturing high-amplitude spikes with finer resolution, significantly improving signal integrity.

The quantization step ranges from 44.8 mV for lower amplitudes to 1.4 mV for higher amplitudes, enabling effective noise suppression in neural recordings. The circuit was designed in a 180 nm CMOS process, resulting in 0.09 mm^2^ area, and was validated by means of post-layout simulations that confirmed the expected performance of the system, including a low power consumption of 62.4 µW. This makes the proposed solution highly suitable for integration into high-throughput neural recording systems. The ADC operates at a sampling frequency of 16 kS/s with a 1 V supply, ensuring efficiency and robustness for real-world biomedical applications.

## Figures and Tables

**Table 1:** Transistors size of the VCO-based ADC core.

List	Width/Length [µm/µm]
M1p-M3p	34/2
M1n-M3n	10/2
M4p-M9p	11/11
M10p-M13p	40/8
M4n-M7n	16/2
M14p-M15p	120/8

**Table 2:** Transistors size of the switches in the calibration section.

List	Width/Length [µm/µm] for S1	Width/Length [µm/µm] for S2 (Each of Array)
M1n	4/0.18	1/0.18
M1p	8/0.18	2/0.18
M2p	2/0.18	0.5/0.18
M3p	8/0.18	2/0.18
M2n	4/0.18	1/0.18

**Table 3:** State-of-the-art neural recording ADCs.

	[20] ^++^	[18] **	[24] ^++^	[13] **	[17] ^++^	[16] **	[14] **	This Work ^++^
Process	0.18	0.18	0.18	0.18	0.18	0.18	0.18	0.18
ADC type	Two-stage, logarithmic SAR ADC	Exponential counter-based ADC with subthreshold-transistor based DAC	Linear, VCO-based	Exponential, SAR	Logarithmic ADC, Log-based DAC	Nonlinear ADC, piece-wise linear SAR ADC	Nonlinear, digital— programmable SAR ADC	Nonlinear, VCO-based
Number of bits	6	8	10	8	3	7	10	8
Reconfigurability	No	No	No	NO	No	Yes	Yes	Yes
Supply (V)	1.8	1.8	1	1.8	1.8	1.8	1.2	1
Sampling frequency (kS/s)	25	5000	25	25	250	42	33	16
Input range (V)	1	0.3	0.16	1	1	1	0.9	1
Power consumption (µW)	14.6	3.11 *	20	87.2	42.7	105	6.3	62.4
Area (mm^2^)	0.164	0.0069	0.027	0.036	NA	0.46	1.54	0.09
*** FOM(J/conv-step)	9.12p	2.43f	0.78p	13.62p	21.35p	19.53p	0.18p	15.22p

* Just the nonlinear DAC is presented in this work. ^++^ simulated only. ** measured results. *** FOM = $\frac{Power}{f_{s}*2^{Resolution}}$.

## Data Availability

The original contributions presented in the study are included in the article, further inquiries can be directed to the corresponding author.

## References

[1] Wells R.E., Baute V., Wahbeh H. (2017). Complementary and Integrative Medicine for Neurologic Conditions. Med. Clin. N. Am..

[2] Testerman R.L., Rise M.T., Stypulkowski P.H. (2006). Stypulkowski. Electrical stimulation as therapy for neurological disorders. IEEE Eng. Med. Biol. Mag..

[3] Jiang W., Hokhikyan V., Chandrakumar H., Karkare V., Markovic D. (2017). A ±50-mV Linear-Input-Range VCO-Based Neural-Recording Front-End With Digital Nonlinearity Correction. IEEE J. Solid-State Circuits.

[4] Zhu B., Shin U., Shoaran M. (2021). Closed-Loop Neural Prostheses With On-Chip Intelligence: A Review and a Low-Latency Machine Learning Model for Brain State Detection. IEEE Trans. Biomed. Circuits Syst..

[5] Amon A., Alesch F. (2017). Systems for deep brain stimulation: Review of technical features. J. Neural Transm..

[6] Shokri R., Koolivand Y., Shoaei O., Aiello O., Caviglia D.D. Multipolar Stimulator for DBS Application with Concurrent Imbalance Compensation. Proceedings of the 2023 30th IEEE International Conference on Electronics, Circuits and Systems (ICECS).

[7] Burkhard P.R., Vingerhoets F.J.G., Berney A., Bogousslavsky J., Villemure J.-G., Ghika J. (2004). Suicide after successful deep brain stimulation for movement disorders. Neurology.

[8] Cheng C.-H., Tsai P.-Y., Yang T.-Y., Cheng W.-H., Yen T.-Y., Luo Z., Qian X.-H., Chen Z.-X., Lin T.-H., Chen W.-M. (2018). A Fully Integrated 16-Channel Closed-Loop Neural-Prosthetic CMOS SoC With Wireless Power and Bidirectional Data Telemetry for Real-Time Efficient Human Epileptic Seizure Control. IEEE J. Solid-State Circuits.

[9] Hartmann C.J., Fliegen S., Groiss S.J., Wojtecki L., Schnitzler A. (2019). An update on best practice of deep brain stimulation in Parkinson’s disease. Ther. Adv. Neurol. Disord..

[10] Tong X., Ghovanloo M. (2016). Multichannel Wireless Neural Recording AFE Architectures: Analysis, Modeling, and Tradeoffs. IEEE Des. Test.

[11] Sharma M., Gardner A.T., Strathman H.J., Warren D.J., Silver J., Walker R.M. (2018). Acquisition of Neural Action Potentials Using Rapid Multiplexing Directly at the Electrodes. Micromachines.

[12] Pazhouhandeh M.R., Kassiri H., Shoukry A., Weisspapir I., Carlen P.L., Genov R. (2021). Opamp-Less Sub-μW/Channel Δ-Modulated Neural-ADC With Super-GΩ Input Impedance. IEEE J. Solid-State Circuits.

[13] Judy M., Sodagar A.M., Lotfi R., Sawan M. (2014). Nonlinear Signal-Specific ADC for Efficient Neural Recording in Brain-Machine Interfaces. IEEE Trans. Biomed. Circuits Syst..

[14] Badami K., Ramos J.-C.P., Lauwereins S., Verhelst M. Mixed-signal programmable non-linear interface for resource-efficient multi-sensor analytics. Proceedings of the 2018 IEEE International Solid-State Circuits Conference—(ISSCC).

[15] Danial L., Sharma K., Dwivedi S., Kvatinsky S. Logarithmic Neural Network Data Converters using Memristors for Biomedical Applications. Proceedings of the 2019 IEEE Biomedical Circuits and Systems Conference (BioCAS).

[16] Sengupta S., Johnston M.L. (2021). A Widely Reconfigurable Piecewise-Linear ADC for Information-Aware Quantization. IEEE Trans. Circuits Syst. II Express Briefs.

[17] Sirimasakul S., Thanachayanont A. A Logarithmic Level-Crossing ADC with Fixed Comparison Window. Proceedings of the 2022 19th International Conference on Electrical Engineering/Electronics, Computer, Telecommunications and Information Technology (ECTI-CON).

[18] Jomehei M.G., Sheikhaei S., Hafshejani E.H., Mirabbasi S. (2022). A Low-Power Logarithmic CMOS Digital-to-Analog Converter for Neural Signal Recording. IEEE Trans. Circuits Syst. II Express Briefs.

[19] Pena-Ramos J.-C., Badami K., Lauwereins S., Verhelst M. (2018). A Fully Configurable Non-Linear Mixed-Signal Interface for Multi-Sensor Analytics. IEEE J. Solid-State Circuits.

[20] Sundarasaradula Y., Constandinou T.G., Thanachayanont A. A 6-bit, two-step, successive approximation logarithmic ADC for biomedical applications. Proceedings of the 2016 IEEE International Conference on Electronics, Circuits and Systems (ICECS).

[21] Shokri R., Koolivand Y., Shoaei O., Aiello O., Caviglia D. A Nonlinear, Low-Power, VCO-Based ADC for Neural Recording Applications. Proceedings of the 2023 5th Iranian International Conference on Microelectronics (IICM).

[22] https://www.niktek.ir/index.php/products/experimental/ndl.

[23] Razavi B. (2017). Design of Analog CMOS Integrated Circuits.

[24] Tong X., Wang J. A 1 V 10 bit 25 kS/s VCO-based ADC for implantable neural recording. Proceedings of the 2017 IEEE Biomedical Circuits and Systems Conference (BioCAS).

[25] Pochet C., Huang J., Mercier P., Hall D.A. (2021). A 174.7-dB FoM, 2nd-Order VCO-Based ExG-to-Digital Front-End Using a Multi-Phase Gated-Inverted-Ring Oscillator Quantizer. IEEE Trans. Biomed. Circuits Syst..

[26] Nguyen V., Schembari F., Staszewski R.B. (2022). A Deep-Subthreshold Variation-Aware 0.2-V Open-Loop VCO-Based ADC. IEEE J. Solid-State Circuits.

[27] Rubino R., Crovetti P.S., Aiello O. Design of Relaxation Digital-to-Analog Converters for Internet of Things Applications in 40nm CMOS. Proceedings of the 2019 IEEE Asia Pacific Conference on Circuits and Systems (APCCAS).

[28] Stanchieri G.D.P., Aiello O., De Marcellis A. A 0.4 V 180 nm CMOS Sub-μW Ultra-Compact and Low-Effort Design PWM-Based ADC. Proceedings of the 2024 IEEE International Symposium on Circuits and Systems (ISCAS).

[29] Della Sala R., Spinogatti V., Bocciarelli C., Centurelli F., Trifiletti A. (2023). A 0.15-to-0.5 V Body-Driven Dynamic Comparator with Rail-to-Rail ICMR. J. Low Power Electron. Appl..

[30] Della Sala R., Centurelli F., Scotti G., Palumbo G. (2024). Rail to Rail ICMR and High Performance ULV Standard-Cell-Based Comparator for Biomedical and IoT Applications. IEEE Access.

[31] Garvi R., Granizo J., Gutierrez E., Medina V., Wiesbauer A., Hernandez L. (2023). A VCO-ADC Linearized by a Capacitive Frequency-to-Current Converter. IEEE Trans. Circuits Syst. II Express Briefs.

[32] Yeon P., Bakir M.S., Ghovanloo M. Towards a 1.1 mm2 free-floating wireless implantable neural recording SoC. Proceedings of the 2018 IEEE Custom Integrated Circuits Conference (CICC).

[33] Wu T.-F., Chen M.S.-W. (2019). A Noise-Shaped VCO-Based Nonuniform Sampling ADC With Phase-Domain Level Crossing. IEEE J. Solid-State Circuits.

[34] Zhao W., Li S., Xu B., Yang X., Tang X., Shen L., Lu N., Pan D.Z., Sun N. (2020). A 0.025-mm2 0.8-V 78.5-dB SNDR VCO-Based Sensor Readout Circuit in a Hybrid PLL-∆∑M Structure. IEEE J. Solid-State Circuits.

[35] Huang J., Mercier P.P. (2021). A 94.2-dB SNDR 142.6-μW VCO-Based Audio ADC With a Split-ADC Differential Pulse Code Modulation Architecture. IEEE Solid-State Circuits Lett..

[36] Hu C.M. (2010). Modern Semiconductor Devices for Integrated Circuits.

[37] Li M.-X., Jiang C.-Y., Pan Y.-Y., Chen H.-H., Hsu Y.-W. Using Inversion-mode MOS Varactors and 3-port Inductor in 0.18-µm CMOS Voltage Controlled Oscillator. Proceedings of the 2019 International Symposium on Intelligent Signal Processing and Communication Systems (ISPACS).

[38] Margalef-Rovira M., Saadi A.A., Vincent L., Lepilliet S., Gaquiere C., Gloria D., Durand C., Barragan M.J., Pistono E., Bourdel S. (2020). Highly Tunable High-Q Inversion-Mode MOS Varactor in the 1–325-GHz Band. IEEE Trans. Electron Devices.

